# Right Heart Phenotype in Heart Failure With Preserved Ejection Fraction

**DOI:** 10.1161/CIRCHEARTFAILURE.120.007840

**Published:** 2021-04-19

**Authors:** Marco Guazzi, Robert Naeije

**Affiliations:** 1 Department of Biological Sciences, University of Milano, Italy (M.G.).; 2 Cardiology Division, San Paolo Hospital, Italy (M.G.).; 3 Erasme Hospital, Free University of Brussels, Belgium (R.N.).

**Keywords:** heart failure with preserved ejection fraction, phenotype, pulmonary hypertension, right heart

## Abstract

The health burden of heart failure with preserved ejection fraction is increasingly recognized. Despite improvements in diagnostic algorithms and established knowledge on the clinical trajectory, effective treatment options for heart failure with preserved ejection fraction remain limited, mainly because of the high mechanistic heterogeneity. Diagnostic scores, big data, and phenomapping categorization are proposed as key steps needed for progress. In the meantime, advancements in imaging techniques combined to high-fidelity pressure signaling analysis have uncovered right ventricular dysfunction as a mediator of heart failure with preserved ejection fraction progression and as major independent determinant of poor outcome. This review summarizes the current understanding of the pathophysiology of right ventricular dysfunction in heart failure with preserved ejection fraction covering the different right heart phenotypes and offering perspectives on new treatments targeting the right ventricle in its function and geometry.

Heart failure with preserved ejection fraction (HFpEF) is a major health problem that accounts for nearly one-half of HF hospitalizations.^1^ This clinical condition originates from an impaired left ventricular (LV) filling due to increased diastolic stiffness,^2^ reduced vascular compliance,^3^ and ventricular-vascular uncoupling associated with aging, hypertension, and obesity.^4^ Therapies aimed at reversing the hypertrophic LV phenotype–related diastolic stiffness and the decreased arterial compliance have been neutral or negative on hard end points shifting the attention to alternative targets of interventions.^5^ The LV backward hemodynamic failure becomes rapidly predominant in the manifestations of the syndrome, and most interest has been recently diverted on the right heart (RH) and the pulmonary microcirculation as mainstay contributors to disease progression.^6,7^ Evidence is being gathered that the study of the RH is of relevance for a diagnostic phenomapping^8^ and for a better understanding of its clinical trajectory.^6^

This review details the current knowledge on HFpEF focusing on the RH phenotypes and discusses the pathophysiological and clinical implications along with the emerging hypotheses for targeting the right ventricle (RV) and its coupling with the pulmonary circulation (Pc) as key interventions for reversing the disease progression and the negative outcome.

## Definition, Prevalence, and Clinical Impact of RH Disease Phenotypes

RH disease in HFpEF is highly prevalent^9^ and prognostic,^10^ yielding to 60% mortality at 2 years when of moderate-to-severe entity.^10^ In a landmark report, Shah et al^11^ developed an HFpEF phenomapping–based model by using an unbiased clustering analysis on dense phenotypic data, processing 67 continuous variables. The phenogroup with RH disease included older subjects with the highest rate of comorbidities and had the worst hospitalization/mortality evolution. Similarly, in a recent machine learning analysis,^8^ the older phenogroup showed a poorer RV function and a higher burden of risk factors, such as atrial fibrillation (AF) and hypertension. In most conditions triggering HFpEF, RH disease develops as a consequence of enhanced left atrial pressure and pulmonary hypertension (PH).^6,12^ Of note, in 10% to 15% of cases of normal LV ejection fraction (EF), RH disease may be the consequence of infiltrative myocardial disease, such as amyloidosis,^13^ a condition requiring appropriate diagnosis and specific clarification as a final step in the diagnostic algorithm, according to the European Society of Cardiology Consensus Statement on HFpEF diagnosis.^1^ Mortality remains elevated irrespective of the etiology and the pre- or postcapillary nature of PH.^14^ Furthermore, given that in HFpEF the left heart dysfunction is typically associated with comorbid conditions, a specific contributory role of inflammation and oxidative stress in the adverse RH chamber remodeling and dysfunction may not be ruled out^15^ but rather represents an intriguing pathogenetic hypothesis to be tested in large numbers and multiple RH disease phenotypes.^16^ Hemodynamic injury and proinflammatory mediators combine with a multitude of gene expression and pathways involved in RV chamber response to injury.^17,18^ In a recent analyses of the PROMIS-HFpEF study exploring the protein biomarker that may support the HFpEF comorbidity-inflammation paradigm by a high throughput technology, Sanders-van Wijk et al^19^ found that systemic inflammation was associated with tricuspid regurgitation and worse RH function. The contribution of multiple pathways and the variable standard of measurements and cutoff for defining the RH disease well explain the heterogeneity observed in prevalence and clinical course itself.^11^ In the subgroup of patients investigated by echocardiography in the PARAGON-HF trial, the rate of RH disease was 30%.^20^ In the large HFpEF cohort of Olmsted County,^9^ RH disease occurred in 35% of cases, as assessed by tricuspid annular plane systolic excursion (TAPSE) measurements, and in 21% of cases by semiquantitative visual score analysis, suggesting a quite weak concordance between the two methods. A similar degree of failure, however, was found if both reduced TAPSE and low motion analysis coincided. RH disease patients were more likely to have AF and to receive higher doses of diuretics. An improved consensus on the diagnostic criteria of RH disease in HFpEF appears to be desirable.

In a study phenotyping RH by assessing RV to Pc coupling by the TAPSE/PASP (pulmonary arterial systolic pressure) ratio,^6^ AF and use of diuretics were consistently associated with the worst RH disease phenotypes. The recurrent link between RH disease development and AF has been confirmed in a number of investigations^21–23^ with some controversy as to whether AF being the cause or the consequence.^16,24^

Although RH disease has been mostly studied in cross-sectional analyses, Obokata et al^23^ recently addressed the time course of functional changes over a median follow-up of 4.0 years. They found a 10% decline in RV fractional area change and 21% increase in RV end-diastolic area. RH disease developed at a rate higher and faster than corresponding changes in the LV and was associated with coronary disease, greater body weight, higher PA and LV filling pressures, RV dilation, and, again, higher rate of AF. Interestingly, RV diastolic dysfunction and increased stiffness occurred before the development of systolic dysfunction, and subjects who developed incident RH disease exhibited a ≈2-fold increased risk of death. Along with these changes, reports document some myocardial fibrosis at the RV insertion points^25^ and even RV diffuse fibrosis as peculiar features in HFpEF.^26^ Interestingly, the myocardial fibrosis at the RV insertion point has been found to correlate with wedge, mean pulmonary, and right atrial pressures.^25^

## Pathophysiology of RH Dysfunction to Failure

**Figure 1.**
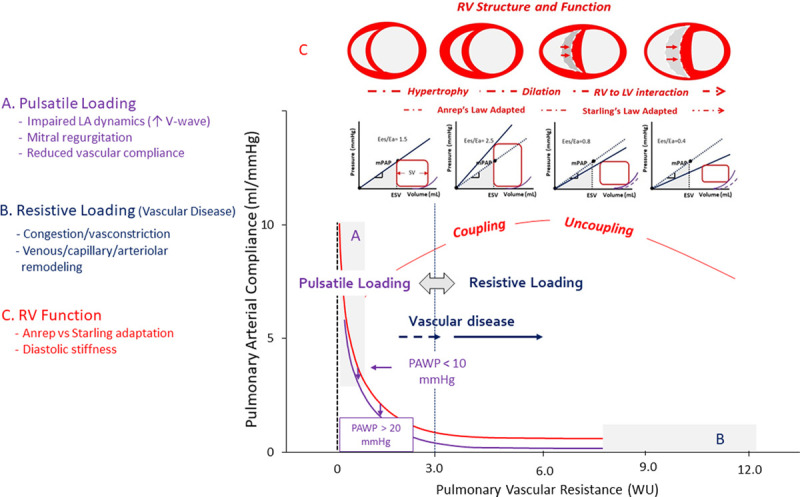
**Evolving stages in right ventricular (RV) geometry and functional changes based on the imposed hemodynamic loading.** Pulmonary vascular resistance (PVR) and pulmonary arterial compliance (PCA) exhibit an inverse relationship described as PVR×PCA time constant, which broadly defines RV impedance. This time constant is affected by pulmonary artery wedge pressure (PAWP) shifting the relationship to the left as typically occurs in left-sided pulmonary hypertension. For PVR <3 WU, changes in time constant are primarily driven by the changes in pulsatile loading (impaired LA dynamics and increase in V wave with or without a specific contribution of mitral regurgitation and vascular venous pathology). Although vascular (veins and capillaries) disease injury (congestion and vasoconstriction) occurs even with normal PVR, for increasing PVR >3 WU, the time constant is highly affected by the resistive loading reflective of the remodeling process. The RV adapts and maladapts to impedance changes according to the Anrep and Starling laws with development of hypertrophy and dilatation. Changes in geometry combine with increased diastolic stiffness, favor tricuspid valve incompetence, and promote the interventricular septum position shift to the left yielding to a progressive loss of contractility and uncoupling with the pulmonary arterial circulation. ESV indicates end-systolic volume; LA, left atrium; LV, left ventricle; and WU, Wood unit.

The RV is a thin-walled volume generator functionally coupled to a low impedance Pc (Figure 1).^27^ As such, the RV is sensitive to brisk increases in pulmonary arterial pressure (PAP), such as in massive pulmonary embolism or experimental PA banding, and may result in acute dilatation and rapidly fatal cardiogenic shock.^28^ However, if given time, the RV adapts to increased afterload by increased contractility thus preserving the Pc coupling and flow output response to metabolic demand. This has been established in experimental and clinical studies in which high-fidelity manometer-tipped catheters were used to derive RV end-systolic elastance (Ees) or end-systolic pressure (ESP) divided by end-systolic volume as gold standard measure of contractility and arterial elastance (Ea) or ESP divided by stroke volume as a validated lumped parameter of afterload.^29^ The Ees/Ea ratio measurement of RV-PA coupling is normally around 1.5. It is preserved during exercise in healthy subjects and is either preserved or decreased at rest but decreases during exercise in severely increased PAP such as in pulmonary arterial hypertension (PAH).^30^ The Ees/Ea ratio has some reserve, as it has to be decreased by some 50%, from 1.5 to around 0.8 until there is an increase in RV volumes. The Ees/Ea decreases to these critical values in pulmonary vascular disease with severe increases in PAP, such as PAH^30^ or chronic thromboembolic PH,^31^ but may already reach uncoupling values in the presence of mild to moderately increased PAP, as has been demonstrated in experimental HF.^32^

Dilatation of the RV signals a shift from homeometric (systolic function) adaptation (Anrep law) to heterometric adaptation (Starling law) to chronic loading conditions.^27^ There is shared information content between the Ees/Ea ratio and EF, as the Ees/Ea ratio can indeed be simplified for a common term of ESP as stroke volume/end-systolic volume, which in turn is equal to EF/(1−EF).^29,33^ An Ees/Ea ratio of 0.8 corresponds to an EF of 35% to 40%.

Practically, RV dysfunction can be defined by a decreased Ees/Ea or EF but preserved end-diastolic volume and end-systolic volume and its transition from adaptive hypertrophy to failure by decreased Ees/Ea or EF and augmented end-diastolic volume and end-systolic volume above normal values.^27^ As reported in the Figure 1, failure would be the turning point from predominantly Anrep to predominantly Starling adaptations of the RV to increased loading. Imaging of RV volumes is, therefore, essential to pick the central steps of this transition process up.

The pressure-volume loop also offers a diastolic elastance curve as a gold standard measure of diastolic function. The diastolic elastance curve has a curvilinearity that increases with increased end-diastolic volume and can be described by an equation that contains a diastolic stiffness coefficient, β. The diastolic stiffness of the RV correlates with disease severity in PAH.^34^ It is conceivable to suspect that the same would be true for HFpEF but this relevant aspect has not been yet systematically detailed in the evolving stages of PH due to HFpEF.^35^

Increased LV filling pressures in HF, as assessed by pulmonary artery wedge pressure (PAWP) measurements, are initially transmitted upstream in a close to 1/1 relationship to mean PAP (mPAP) with unchanged pulmonary vascular resistance (PVR). Even with relatively normal PVR at rest, patients with HFpEF display a compromised pulmonary arterial compliance (PAC)^36^ as a consequence of an increase in the pulsatile loading, which typically occurs in the presence of atrial myopathy and increased V wave, especially when associated with AF^37^ and atrial functional mitral regurgitation.^38^ The progressive impairment in LA dynamics becomes a central player in the pulmonary congestion cascade and a limiting step in the lung fluid clearance process favoring perivascular inflammatory reaction.^36,39^

All together, these mechanisms promote the time- and pressure-related pulmonary vascular remodeling and increase the gradient between PAWP and mPAP.^27^ Stiffening of the pulmonary vessels, starting from the venous system, further increases this gradient.

One could think of quantifying RV afterload by a PVR calculation, assuming Ea or ESP/stroke volume to be equal to PVR divided by heart rate and mPAP a satisfactory estimation of RV ESP. However, this approach is misleading because of variable underestimation of ESP by mPAP.^40^ RV afterload is determined by a dynamic interplay between PVR, PAC, and wave reflection.^41^ Although specific evidence in HFpEF patients is lacking, a number of studies have shown that wave reflection affects PA pressure curves by late systolic peaking of pressure and PA flow curves by late or midsystolic deceleration of flow.^42^ However, these morphological aspects do not necessarily impact on RV afterloading as the product of PVR and PAC or so-called time constant of the Pc (RC time) remains constant or nearly constant, allowing for an essentially unchanged oscillatory to steady flow work (Wosc/Wst) ratio of 25% in all PH conditions.^43^ Extreme reported variations of the RC time still do not prevent valid estimation of RV afterload by a total hydraulic load calculation (Wtot) as 1.2 to 1.4× Wst.^44^

This being said, the constant value of the product of PVR by PAC implies a hyperbolic relationship so that the relative contribution of PAC to afterload is greater in case of increased PAWP with normal or only mildly elevated mPAP. This may at least, in part, explain greater predictive capability of outcome of PAC versus PVR in HF patients and especially in HFpEF.^45^ However, whether a decreased PAC because of increased PAWP or altered left atrial structure and function may be sufficient to increase Wosc/Wst in HF patients remains to be established.

RV distension is associated with functional tricuspid regurgitation from stretching of the tricuspid valve annulus. Tricuspid valve annulus dilates along the anterior and posterior aspects because of the relatively fixed portion of the medial aspects of the annulus. With this distension, there is a change in the shape of the valve with flattening and modifications of the normal papillary muscle to leaflet and valve relationship.^46^ The volume load of the tricuspid regurgitation can itself lead to further increase in RV dilatation and associated preload-induced increase in afterload as defined by RV wall tension. Moderate-to-severe tricuspid regurgitation is quite common in stage B and C HFpEF, and^47^ geometric changes in tricuspid valve and associated tricuspid insufficiency hold a considerable impact on survival.^48,49^

**Figure 2.**
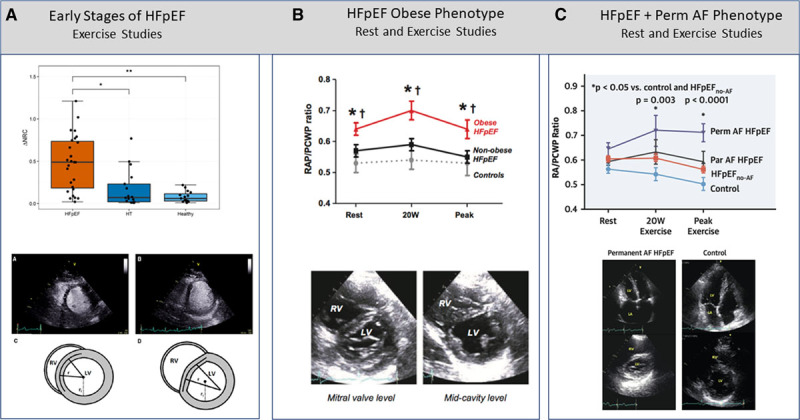
**Studies on right ventricular (RV)–left ventricular (LV) diastolic ventricular interaction in heart failure with preserved ejection fraction (HFpEF).** The increase in RV volume and pressure induced by exercise shifts the interventricular septum shifts toward the LV, distorting RV morphology, impairing filling, and decreasing contractile efficiency. The downstream effects of this sequence of events consist in an increased transmural pressure favored by the pericardium constraint. **A**, Early HFpEF studied during exercise. Reprinted from Parasuraman et al^53^ with permission. **B**, Obese HFpEF phenotype at rest. Reprinted from Obokata et al^4^ with permission. Copyright ©. **C**, Permanent atrial fibrillation (Perm AF) HFpEF phenotype as investigated at rest. Reprinted from Reddy et al^37^ with permission. Copyright © 2020, Elsevier. AF indicates atrial fibrillation; HT, hypertension; PCWP, pulmonary capillary wedge pressure; and RAP, right atrial pressure.

As a result of the increase in RV volume and pressure, the interventricular septum shifts toward the LV, further distorting RV morphology and contractile efficiency, and an unfavorable ventricular interdependence dominates the impaired biventricular mechanics and hemodynamic perturbation.^50^ The pericardium primarily contributes to the RV to LV interaction exerting a constraining effect.^51^ Ventricular interdependence is diastolic, which alters LV diastolic function by competition for space between the LV and the dilated RV within a nondistensible pericardium,^51^ but also systolic, which to some extent preserves RV contractility by the entrainment effect of preserved LV systolic function.^22^ In the end, negative diastolic interdependence may prevail, resulting in a low-cardiac-output state by decreasing LV distensibility and preload. Although these step-by-step variations in RV geometry and function have been well described in early HFrEF stages, there is now clear characterization in HFpEF.^52^ Specifically, in HFpEF, the role of an unfavorable diastolic ventricular interaction has been elegantly unmasked even in the early stages during exercise^53^ (Figure 2A); it becomes a mainstay mechanism of further PAWP increase and capillary injury in the obese phenotype (Figure 2B)^4^ and is exacerbated by the coexistence of permanent AF (Figure 2C).^37^

The effects of pressure-induced RV failure and related changes in molecular pathways of myocyte dysfunction in the context of HFpEF remain poorly investigated certainly representing a field of high interest for advancing knowledge and gaining insights on the new therapeutic advancements. Several neurohormonal and citotoxic pathways play a central role in the response to loss of RV function and chamber geometry. The remodeling process impairs the coronary flow reserve, and ischemia triggers myocardial downstream effects such as fibrosis and collagen hyperproliferation.^16^ Starting from the leading concepts that enhanced oxidative stress, fibrotic, hypertrophic, and inflammatory signaling and depressed NO and endoplasmic reticular signaling are typical of HFpEF, a recent analysis performed in 63 HFpEF undergoing RH catheterization with RV endomyocardial biopsy has given direct information on the RV myocyte transcriptomic signatures. mRNA analyses established and confirmed altered gene expression in pathways often postulated as central to HFpEF pathogenesis including fibrosis, hypertrophy, oxidant stress, and inflammation with similar expression for both RV and LV myocytes.^54^ A recent analysis of RV systolic sarcomeric function combined with myocyte stiffness has shown a higher depressed RV systolic function but less passive stiffness in the HFpEF obese phenotype compared with the primarily hypertensive/hypertrophic phenotype.^55^ These findings may help explain the worse outcomes in obese HFpEF and prospect toward newer approaches to treatment with sarcomere stimulators.

A contributory role to RV function impairment and elevated right-sided filling pressure has been recently documented by epicardial adipose tissue,^56^ further confirming the importance of the inflammatory pathogenesis of RV dysfunction in HFpEF.

## Imaging Assessment of RV Geometry, Systolic Function, and Coupling With the Pc

**Figure 3.**
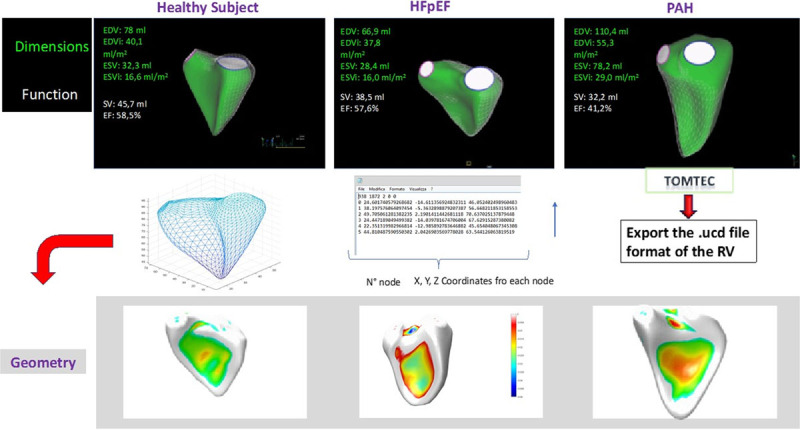
**Three-dimensional right heart echocardiography reconstruction and analyses** Dimensional, functional, and geometric data of a healthy subject (**A**) versus a heart failure with preserved ejection fraction (HFpEF; **B**) and a pulmonary arterial hypertension (PAH) patient (**C**). Postprocessing analysis of nodes and colorimetric scale reconstruction of the right ventricular (RV) shape intriguingly shows that the main element in the normal RV function is the midposition of the septum with early HFpEF compared with control already shows some bulging of the septum in the left ventricle which is actually typical of PAH condition. EDV indicates end-diastolic volume; EDVi, end-diastolic volume indexed; EDSi, end-systolic volume indexed; EF, ejection fraction; ESV, end-systolic volume; SV, stroke volume.

Historically, imaging of RV structure and function was considered challenging because of the unfavorable geometry, limited definition of endocardial surface, and peculiar contraction pattern. The development and introduction in clinical practice of advanced imaging and laboratory-controlled hemodynamic studies allow now to perform full studies on the RH and gain the most relevant information.^57^ Magnetic resonance imaging is the gold standard for assessment of RV volume, geometry, and systolic function, but 3-dimensional reconstruction by echocardiography, with post processing analysis of nodes and colorimetric scale, is certainly a step forward for monitoring the parallel evolving changes in dimensions, function, and geometry (Figure 3). However, case series studies^9,22,58–62^ to date performed in HFpEF patients, except for few ones,^63^ have analyzed RV function by different parameters obtained with 2-dimensional and TDI echocardiography, and a comprehensive comparison of the best accuracy of available imaging metrics of RV structure and function is lacking. In most studies, the predominant measure used for quantifying systolic function has been TAPSE with some other poorly sensitive but clinically meaningful measures of geometry, such as right atrial and ventricular area.^9,22,58–62^ It is noteworthy that a quite inaccurate measure of RV remodeling such as free wall thickness obtained at echocardiography was the strongest pathophysiological predictor of adverse outcome in a large population of patients with HFpEF.^59^ Especially, the combination of RV free wall thickness and TAPSE was prognostically useful for overall outcome and HF hospitalization prediction, respectively, and their prognostic utility was not attenuated by adjustment of HF severity. There was also an interaction between RV wall thickness and LV mass index in predicting composite outcome.

The study of RV systolic dysfunction in HFpEF has recently expanded from use of simple indicators of RV shortening or systolic velocities to measures of RV contractile state.^60^ This is of paramount importance considering that indexes of systolic shortening are highly sensitive to pressure load, which is typically elevated in the presence of PH-HFpEF, making it difficult to discern whether RV dysfunction is reflective of afterload mismatch or intrinsic myocardial disease or both. A thorough evaluation of RV contractility by pressure-volume analysis with measures of maximal elastance has been applied to the study of RH function in HFpEF primarily in experimental conditions^64^ or isolated human cases.^35^

The search of a noninvasive, easy-to-apply, and clinically useful indicator of RV contractility has brought to the application and proposal of simple echocardiography-derived measure of coupling such as by plotting the relationship of a measure of RV shortening (TAPSE) to the developed force (PASP).^60^ At variance with the relationship, the ratio of TAPSE/PASP was assumed to broadly inform about RV-PA coupling, with TAPSE considered as a load-dependent surrogate of Ees and PASP as an indirect estimate of Ea, and as such showed to be of high prognostic relevance in HFpEF.^60^ Studies have confirmed its clinical and prognostic capability, not only in HF^65^ but also in PAH and in acute pulmonary embolism.^66–67^ In 2 studies of group 2 PH including HFpEF patients, categorization according to TAPSE/PASP inversely distributed in the hyperbolic relationship of PAC versus PVR.^6,68^

**Figure 4.**
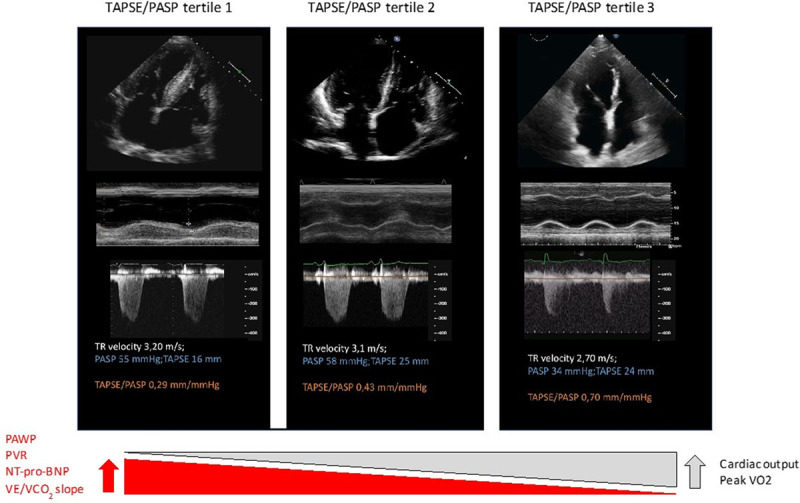
**Representative cases of right ventricular (RV) dysfunction and RV–pulmonary circulation coupling phenotyping by tricuspid annular plane systolic excursion (TAPSE)/PASP ratio according to tertiles: 1, <0.35; 2, between 0.35 and 0.57; 3, >0.57.** A cutoff of 0.36 has been documented by most studies as the most sensitive cutoff for prognostic definition. NT-proBNP indicates N-terminal pro-B-type natriuretic peptide; PASP, pulmonary arterial systolic pressure; PAWP, pulmonary artery wedge pressure; PVR, pulmonary vascular resistance; TR, tricuspid regurgitation; VCO2, carbon dioxide production; VE, ventilation; and VO2, oxygen consumption.

The normal TAPSE/PASP ratio is of 1.2 (range, 0.8–1.6) mm/mm Hg and decreases slightly with aging.^69^ Interestingly, in PH-HFpEF, a TAPSE/PASP ratio of 0.36 discriminated pre- from postcapillary PH in patients with HF,^70^ and, based on these observations, TAPSE/PASP ratio has been used for screening different phenotypes of RV-Pc uncoupling in a parallel assessment of clinical characteristics, pulmonary and systemic hemodynamics, and outcome in HFpEF according to tertile group definition (Figure 4). Progressively increasing levels of natriuretic peptides, worse systemic and pulmonary hemodynamics, abnormal exercise aerobic capacity, and ventilatory inefficiency were observed from the highest to the lowest tertile.

**Figure 5.**
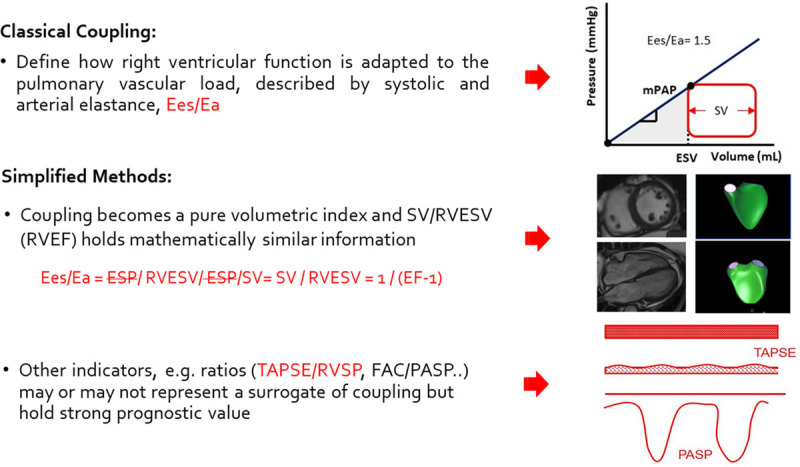
**Differences and clinical value of classical measures of right ventricle–pulmonary circulation coupling (end-systolic elastance [Ees]/arterial elastance [Ea]) and simplified surrogates (ejection fraction [EF] and ratios).** ESP indicates end-systolic pressure; ESV, end-systolic volume; FAC, fractional area change; mPAP, mean pulmonary arterial pressure; PASP, pulmonary artery systolic pressure; RVEF, right ventricular ejection fraction; SV, stroke volume; and TAPSE, tricuspid annular plane systolic excursion.

Echocardiography-derived approaches similar to the TAPSE/PASP ratio have been developed such as, for example, relating RV fractional area changes (%) to mPAP.^22^ The analysis of this relationship showed a downward shift in the PH-HFpEF with AF compared with HFpEF free of AF unmasking some further degree of RV function mismatch due to a partially load-independent fashion. Other simplified surrogate approaches including RV EF (CMR), (fractional area change)/PASP are clinically relevant,^21,22,71^ and more comprehensive and precise indicators such as the tissue Doppler S′ and the RV free wall strain appear promising. Nonetheless, TAPSE/PASP has been the first approach proposed and to date the only noninvasive echocardiography-derived indicator tested against invasive gold standard measures of Ees/area^6^ and Ees/Ea.^66^ Figure 5 summarizes the established differences and clinical validity of measures of RV-Pc coupling.

In most recent years, there has been a progressive need to recognize and explore the Pc and the RV as an integrated functional unit also during exercise and fluid loading especially in subjects with preserved LV EF and presenting with uncertain diagnosis of true HFpEF. A fluid challenge may be considered as a valid though less strenuous mean than exercise, to test RV function mainly through an increased systemic venous return.^72^ At present, there are no data yet on the effects of fluid loading on RV-Pc coupling in HF. In healthy subjects, exercise is associated with a preserved RV Ees/Ea^30^ but a decreased TAPSE/PASP, which is accentuated with aging. Exercise decreases RV Ees/Ea in PAH, and, most recently, the same has been shown in HF. In that study, Ees/Ea was recalculated from RV pressure waveforms and was typically most decreased in patients with increased PVR. More data are needed to evaluate the clinical relevance of RV-Pc coupling measurements in fluid loading or exercise stress conditions in HFpEF.

## Therapeutic Perspectives

Since the RH is quite sensitive to an increase in afterload due to the elevated left atrial pressure, the first goal of therapy should be to reduce LV filling pressure with the double aims of (1) modulating the abnormal pulsatile loading on the RH and (2) avoiding fluid swelling from pulmonary capillaries to the interstitium with consequent increase in transmural arteriolar pressure and loss of PAC.^39^ Specifically, therapeutic strategies should be aimed at contrasting the multifold pathways involved in lung capillary stress failure and remodeling,^73^ potentially targeting those that may embrace some combinatory biological effects on the pulmonary endothelium with a reverse remodeling of RV myocyte hypertrophy and extracellular matrix proliferation.^74^

Effective decongestion is a must, and methods to obtain a long-lasting effect remain an important challenge with recent evidence pointing on devices to monitor intracardiac and pulmonary pressures.^75^

Andersen et al^58^ performed a prospective invasive hemodynamic and echocardiographic study uniquely examining resting RV and pulmonary vascular function along with RV-Pc coupling responses to acute β-adrenergic stimulation with dobutamine in patients with HFpEF at earlier stages versus controls. RV systolic and diastolic function were impaired in HFpEF subjects compared with controls using both invasive and noninvasive indices. Dynamic RV-Pc coupling analysis, assessed by either invasively determined dP/dt versus mPAP or semi-invasively by the echocardiography of tricuspid annulus tissue Doppler velocity s′ versus mPAP, suggested that RV systolic function was blunted during β-adrenergic stimulation in HFpEF and consequent pulmonary vasodilation compared with controls. These findings indicate that an altered pulmonary arterial tone is a major component of the syndrome, even at early stages when any increase in PAP is simply related to the backward passive effect of increased left atrial pressure. This evidence challenges the appropriate timing for interventions and the rationale of eventually using vasodilator agents.

Intriguingly, the lack of an inotropic positive response during β-adrenergic stimulation could prospect and reinforce the hypothesis that as for the LV, the failing RV would present with β-adrenergic receptor desensitization. However, no specific data in HFpEF patients are available.

Although efforts to effectively treat RV-Pc uncoupling in HFpEF are increasing,^61,76^ historical studies performed with pulmonary vasodilators in HFpEF group 2 PH have not been encouraging and mostly negative.^77,78^ Nonetheless, study hypotheses, design, inclusion criteria, and phenotyping of populations have been quite incomplete, likely contributing to some delay in the search of effective compounds for RH dysfunction due to LV disease. Specifically, the most crucial questions that have been addressed only in part in the past are: which HFpEF phenotype may be more responsive to pulmonary vasodilators; how much to obtain RH unloading as compared with the biological effects; what pathways to target for expecting some reverse remodeling.

In a study of HFpEF with precapillary PH, the effects of PDE_5_ inhibition on both pulmonary hemodynamics and RV function were tested enrolling patients by a prespecified phenotyping process, that is, documented significant LV hypertrophy; RV-LV interaction ratio of RH (central venous pressure) to left heart (PAWP) filling pressures >1; high PVR, increased elastance and documented pulmonary capillary damage (low DLco and alveolar membrane component).^61^ Sildenafil was effective on pulmonary hemodynamics, yielding to a significant RV-Pc recoupling and gas diffusion restoration. These findings have been recently confirmed by Belyavskiy et al^79^ in similar patients of combined pre- and postcapillary PH-HFpEF showing an improvement in RH hemodynamics and exercise performance.

The PASSION trial is underway and will test the effects of tadalafil on hard end points in a larger group of similarly phenotyped patients. The same potential for benefits of PDE_5_ inhibition has not been documented for isolated postcapillary PH-HFpEF,^80^ and the RELAX trial exclusively based on estimated PASP led to neutral results as well.^81^

Overall, the evidence available to date points to the concept that a thorough RH phenotyping is preliminary to any further reasoning in the potential use of PDE_5_ inhibitors in PH-HFpEF (Table) and conceivably explains differences across studies.

**Table.**
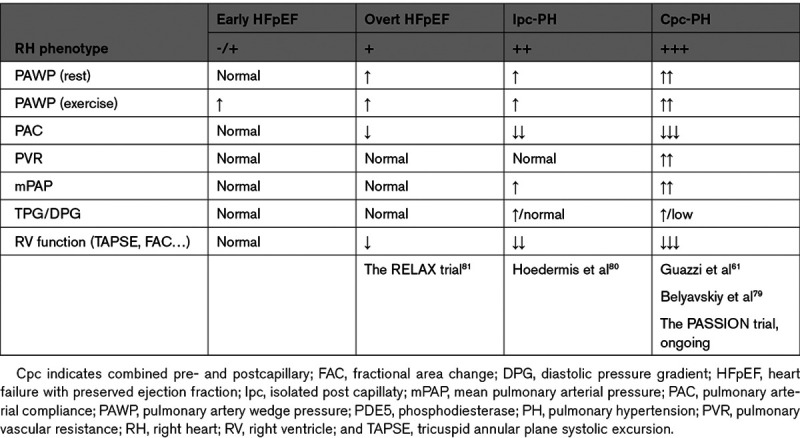
Studies investigating the effects of PDE_5_ inhibition on the different right heart phenotypes from early HFpEF to advanced PH-HFpEF

Recently, treatment with β3AR (beta-3 adrenergic receptor) agonists has been found to improve pulmonary hemodynamics, RV remodeling, and pulmonary vascular proliferation in a translational porcine model of postcapillary chronic PH.^82^ In humans, findings with albuterol in HFpEF have shown an improvement in exercise pulmonary vascular reserve without worsening left heart congestion.^83^ Interestingly, some of the benefits observed with albuterol were driven by a reduction in LV transmural pressure suggesting an RV unloading effect due to perivascular and peribronchial fluid reabsorption and an RV unloading effect.

Similar findings have been described during acute administration of inhaled sodium nitrite showing a reduction in biventricular filling pressures and PAPs at rest and during exercise in HFpEF.^84^

An ongoing study of combined pre- and postcapillary PH-HFpEF patients, the SPHERE HF, will test the effects of a β3AR, mirabegron, on RH performance using advanced imaging techniques (CMR and 3-dimensional echocardiography) and several markers of subclinical myocardial disease (eg, speckle tracking–derived strain and extracellular volume quantified by T1 mapping CMR) to address even subtle changes in RV function.^85^

Promising results have been observed with the TRPV4 (transient receptor potential vanilloid 4) channel, which regulates fluid transit across the alveolar capillary interface and represents a novel target to reduce lung interstitial and perivascular water, independent of pulmonary capillary hypertension, definitively impacting the RV.^86^

An interesting nonpharmacological approach to the treatment of HFpEF is the interatrial communication device whose indications to implantation require no PH at rest and normal RV function. Whether this approach may delay or prevent development of RV dysfunction is a matter of interesting investigations,^87^ but there is some concern on the temporal-related occurrence of RV dysfunction as suggested by experimental models.^88^

## Conclusions and Perspectives

HFpEF is a complex disease at high and increasing prevalence with no effective therapeutic options. Among the pathophysiologic abnormalities that contribute to the heterogeneity of HFpEF syndrome, recent epidemiological and clinical studies have progressively focused on the RH, pointing out its basic role as a mediator of the progression of the disease, exhibiting a high prediction value for clinical worsening, and representing a main target of newly developed therapeutic opportunities. Several clinical RH phenotypes at rest and during physical challenge have been described, and the pathophysiology behind the different evolving stages is a matter of intriguing investigation. The main reason for the development of RH dysfunction is the augmented load imposed by the increased left atrial pressure associated with LV diastolic dysfunction and atrial myopathy, yielding to an increased pulsatile loading. The development of pulmonary vascular disease and wall remodeling further exacerbates the RH impedance through an increase in resistive load.

The gold standard for measuring RH systolic function and its coupling with Pc are cardiac and vascular elastances measured by pressure-volume loops, but a number of echocardiography-derived parameters are alternatively used in daily practice for acquiring either functional and morphological information. Although these measures reflect, only in part, the complex hemodynamic adaptations of the RH, the functional assessment is now comprehensively approached through advanced imaging along with simplified method of RV-PC coupling. These indicators demonstrate a remarkable prognostic value starting from the earlier stages of the disease. Based on this evidence, most of the future research should focus on how the study of RH phenotypes could implement our knowledge on HFpEF.

Upcoming research in the field should aim at a better understanding of the mechanisms involved in the RV geometric and functional progression starting from specific attention on the time-dependent changes in LA dynamics and deepest knowledge on the pathways involved in the lung vessel injury and remodeling processes.

## Sources of Funding

This study was supported by Monzino Fundation, Milano, Italy.

## Disclosures

None.
